# Recreational activities and psychological distress during the COVID-19 crisis: a cohort study from Norway

**DOI:** 10.1186/s12889-025-22942-7

**Published:** 2025-05-31

**Authors:** Rebecca Dyer Ånensen, Viggo Krüger, William Hazell, Gro Mjeldheim Sandal, Lars Thore Fadnes

**Affiliations:** 1 Section for Analyses, Department of Business Development, Culture and Sports in the City of Bergen, Bergen, Norway; 2 Department of Sociology, Faculty of Social Science, University of Bergen, Bergen, Norway; 3 Department of Music, the Grieg Academy, University of Bergen, Bergen, Norway; 4 Department of Psychosocial Science, University of Bergen, Bergen, Norway; 5 Department of Global Public Health and Primary Care, Faculty of Medicine, University of Bergen, Bergen, Norway; 6 Department of Addiction Medicine, Bergen Addiction Research, Haukeland University Hospital, Bergen, Norway

**Keywords:** Cohort study, Recreational activities, Cultural audience, Physical activity, Psychological distress, COVID-19, Norway

## Abstract

**Background:**

This study aims to assess the relationship between engaging in recreational activities *before* the pandemic and levels of psychological distress *during* the pandemic.

**Methods:**

Data from a cohort study (*n* = 16,212) collected at three time points (April 2020, January 2021, and May 2022) in Bergen, Norway, were analysed via a linear mixed model. Associations between psychological distress and five dimensions of recreational activities were assessed, both at baseline and over time.

**Results:**

Regular physical activity before the pandemic was linked to lower psychological distress at baseline, whereas for cultural audience participation, this relationship was inverted. Over time, regular cultural audience participation before the pandemic was associated with a slight reduction in psychological distress. No clear baseline associations or significant changes over time were found for cultural practitioner, social networking, or volunteering/religious activities.

**Conclusions:**

These findings suggest that there are links between recreational activities and psychological distress during a crisis, with cultural audience participation possibly reducing distress over time.

**Supplementary Information:**

The online version contains supplementary material available at 10.1186/s12889-025-22942-7.

## Background

Psychological distress, which refers to general symptoms of anxiety and depression, can be seen as a normal fluctuating response to different stressors that is experienced by most people [1]. However, persistent and severe psychological distress may indicate the presence of clinically diagnosed mental health disorders [1]. Psychological distress has also been linked to increased risk of developing somatic conditions like cardiovascular disease and arthritis [2, 3] and higher mortality rates [4]. For these reasons it is important to explore ways to mitigate the impact of psychological distress.

Recent research highlights beneficial effects of recreational activities on mental health. Defined as freely chosen and intrinsically satisfying leisure pursuits, recreational activities can encompass a broad range of activities, from arts and cultural participation to sports and physical activities [5]. Numerous studies link physical activity to positive mental health outcomes [6, 7], with some suggesting it can protect against future psychological distress [8]. Additionally, there is growing evidence of the positive effects of cultural and arts engagement on well-being [9, 10], and in the prevention, management, and treatment of mental illnesses [11]. A Norwegian longitudinal study found that cultural activities are strongly associated with better health, higher life satisfaction, and reduced anxiety and depression [12], which are the main components of psychological distress.

The COVID-19 pandemic significantly impacted both mental health outcomes and access to recreational activities, creating an opportunity to explore the link between these two areas more closely. The pandemic has led to increased levels of psychological distress globally. Social isolation and restricted access to community activities, including recreational activities, were among the reasons for this increase [13]. Few studies have explored the link between a broader range of recreational activities, including physical, cultural, volunteering, and religious activities, and psychological distress during the COVID-19 pandemic, especially using a longitudinal design [13], as we will do in this article.

This study explores the connection between regular engagement in different types of activities before COVID-19 and levels of psychological distress during the pandemic. Following the same respondents over three timepoints during the pandemic, we investigated whether there were significant differences in the levels of psychological distress at baseline and over time between those who reported having regularly engaged in different types of recreational activities before the pandemic and those who did not. To capture which aspects of the activities might be associated with levels of psychological distress, we grouped the different activities into five dimensions of recreational activities: “physical activity” (performing physical activity weekly), “cultural practitioner” (weekly active involvement in cultural activities such as singing in a choir), “cultural audience” (such as attending concerts or theatre events monthly or more), “social networking” (performing recreational activities together with others on a weekly basis, such as playing team sports or singing in a choir) and “volunteering and religious activities” (on a weekly basis).

These connections are worth exploring on the one hand because they can indicate whether different types of participation contribute to people’s psychological robustness in the face of disaster. On the other hand, they can indicate the importance of these types of activities in people’s lives, as many of them were difficult to practice during the pandemic. Thus, this study evaluates the links between participation in different recreational activities before the COVID-19 pandemic and levels of psychological distress during the pandemic, adjusted for other confounding factors.

### Theoretical perspectives

There are several theoretical perspectives that can be helpful in explaining the link between recreational activities and psychological distress in the context of the pandemic. On an individual level, one can look at this link through the lens of coping mechanisms. Effective coping helps maintain an optimal level of activation by either addressing and altering the sources of stress (“problem-focused coping”) or regulating emotional responses to stress (“emotion-oriented coping”) [14]. Emotion-oriented coping refers to a set of strategies that individuals use to manage their emotional responses to stressful situations rather than directly addressing the source of stress. This coping style is particularly significant and prevalent in scenarios where individuals perceive that they have little control over the situation, as was evident in the early stages of the COVID-19 pandemic [15, 16]. While such strategies may encompass various activities, such as listening to music, engaging in creative pursuits, or participating in sports, a common characteristic is their ability to help individuals process emotions and offer temporary relief from stress [17].

One can also look at the connection between recreational activities and psychological distress through a more collective lens. The phenomenon of “collective effervescence” was first conceptualized by French sociologist Émile Durkheim as the synchronization of emotions during collective rituals, ceremonies, and demonstrations [18]. According to Durkheim, collective effervescence represents an intense emotional experience where individuals, through shared actions, develop a unified sense of belonging and group identity. We may connect collective effervescence to the concept of collective coping in the stress literature [19, 20]. Collective coping refers to the strategies and efforts used by a group to manage stress, adversity, or challenges together and is commonly observed in communities, organizations, or social groups during times of crisis [21]. During the pandemic, collective coping included a broad range of both problem-focused and emotion-focused behaviours. These ranged from making sense of the situation, fundraising for research to following restrictions on social contact and providing arenas of emotional support. A Norwegian study suggested that the emphasis on collectively handling the pandemic seemed to make restrictions on individual freedom easier to accept [22]. Attending or actively participating in recreational activities such as cultural or sport events are all possible manifestations of collective, emotion-oriented coping. The shared experience inherent in collective coping can create a sense of solidarity, emotional connection and mutual support, all of which contribute to buffering the negative effects of stressors [23].

## Methods

### Study characteristics: design, population, data collection and study sample

This is a cohort study presenting data from the Bergen-in-change study [24]. A random sample of 81,170 adults from a population of 224,000 in Bergen, Western Norway, were asked to participate in a survey evaluating the effects of the COVID-19 pandemic and the nonpharmaceutical interventions adopted. The sample was representative of the general population in terms of age and sex. The participants were randomly selected from a contact list by the Norwegian Digitalization Agency. Using the web-based platform SurveyXact, the survey was distributed to the invited individuals. At the first time point, in April 2020, 29,535 (36%) of those invited agreed to participate in the study, and of those, 55% (n_0_ = 16,212) completed items of interest for the present paper. Data were collected approximately one year later (9 months after the first assessment), in January 2021 (n_1_ = 16,211), and two years later (25 months after the first assessment), in May 2022 (n_2_ = 8,282). The variability in response time between the individuals for each of the time points was plus/minus a week (about 2 weeks range).

### The Norwegian context during the pandemic

At the first time point of the survey (April 2020), four to six weeks after the first wave of the pandemic, several restrictions due to COVID-19 had already been initiated. Schools and kindergartens were shut down, working from home was encouraged where possible, bars and restaurants were closed, all cultural events were cancelled or postponed, organised sports and other recreational activities were closed, as well as fitness centres and pools. Use of public transport was discouraged. There were also bans on travel and strict quarantine rules for those returning to the country. Social gatherings were restricted to 5 people, and it was recommended to keep a social distance of 2 m with people outside the household [25]. From the end of April, the government started to ease up on the restrictions, and by mid-June 2020 all educational institutions were reopened, public events were allowed for groups up to 200 people and recreational activities for children were exempt from the social distancing rules [25].

In the fall of 2020, there was a second wave of infection, and the government reintroduced some restrictions [26]. Even though vaccination of the population had been initiated by the end of the year [27], January 2021 started with a two week lock down to curtail the increase in infections associated with holiday travel. This meant closing schools and cancelling indoor sports and cultural activities, as well as enforcing social distancing rules [28]. It was around this time the data from the second round of the survey was collected.

In the spring and fall of 2021 there was a third and fourth wave of infection caused by different mutations of the virus. By the end of 2021, 70% of the population was fully vaccinated, and the impact of the new omicron mutation seemed to be less than previous types. In February 2022 all restrictions related to the pandemic were lifted [26]. The data from the last round of the survey was collected in May 2022.

### Questionnaire and study variables

The survey included questions regarding demographic data and many areas of life and health during the initial COVID-19 lockdown. In the analysis for this study, the following background variables were included: age, sex, highest education level achieved, and household income. Survey items related to this study assess participation habits in recreational activities before the COVID-19 pandemic and symptoms of psychological distress at baseline and over time (see Table A1 in Additional file 1 for full description of survey items).

The main outcome variable is psychological distress. Psychological distress was evaluated via the 10-item version of the Hopkins symptom checklist (SCL-10), which examines psychological distress symptoms during the previous week, with the threshold for significant psychological distress set at a mean SCL score of 1.85 [29]. While SCL10 includes separate scales for depression and anxiety, there is a high comorbidity in symptoms [30]. For this study, an overall score for psychological distress was seen as sufficient. Typically, the SCL10 is reported as mean item score, but to make it more easily interpreted by those not very familiar with the scale, we have re-scaled it to a proportional scale from 0 to 1 where 0 is no psychological distress (corresponding to mean item score of 1) and 1 is the maximum level (corresponding to mean item score of 4) of psychological distress. The score is thus to be interpreted as percentage of the maximum.

The different types of recreational activity asked about in the questionnaire are organized sports; individually organized sports/exercise; going for a walk; organized song, music, theatre, or dance; other organized cultural activities; hobby activity/arts and crafts done alone or with friends; going to a museum or an arts exhibition; going to a concert or theatre performance; going to the cinema; going to the library; participating in volunteer work in health, social work or the like; and participating in activities in a religious community, including religious meetings. The activity questions were included at the second time point of the survey (January 2021), asking the participants how often they engaged in these activities before COVID-19. For each of the activities they were given the following alternatives: did not engage in this before COVID-19; less than once a month; 1–3 times a month; every week; and daily. The variables were recoded into dummy variables where one equals participating in the activity every week or daily, and the rest equal zero. The exceptions here are the cultural audience variables (going to the museum/art exhibition/concert/theatre/cinema/library), which were set to 1–3 times a month or more because these are activities that very few people perform on a weekly or daily basis. To reduce complexity and highlight specific aspects of the activities, they were grouped into five dimensions: physical activity, cultural practitioner, cultural audience, social networking, and volunteering and religious activities (Table 1).

**Table 1:** Description of activity dimensions

Dimensions	Criteria	Activities
Physical activity	Activities where movement and exercise are the focus	Organized sports Individually organized sports/exercise Going for a walk
Cultural practitioner	Activities where you practice or produce creative and cultural expression	Organized song, music, theatre, or dance Other organized cultural activities Hobby activity/arts and crafts done alone or with friends
Cultural audience	Activities where you consume creative or cultural expression	Going to a museum or an arts exhibition Going to a concert or theatre performance Going to the cinema; going to the library
Social networking	Activities where you are part of a social group that extends beyond your closest friends and family	Organized sports Organized song, music, theatre, or dance Other organized cultural activities Participate in volunteer work in health, social work or the like Participate in activities in religious community including religious meetings
Volunteering and religious activities	Social activities with a charitable or religious aspect	Participate in volunteer work in health- or social work, Participation in religious activities

*Activities could be included in more than one dimension

### Statistical analyses

The software Stata SE 16 (StataCorp, College Station, TX, USA) was used for all the statistical analyses. The threshold for statistical significance was set to *p* < 0.05 for all analyses unless otherwise stated. In all analyses, we defined time as years from baseline. Linear mixed model analyses were used to investigate whether participation in different activity dimensions before COVID-19, when adjusted for sociodemographic factors, was associated with psychological distress at baseline and to what extent it was associated with any changes in the outcome variable over time. The estimator was set to maximum likelihood. To explore whether the predictors predicted changes in outcome, the interactions between all the independent variables and time were added to the model. We tested the linear mixed model both as a random intercept fixed slope regression model, and with random slopes grouped by time to see if time trends varied between individuals. There was close to no difference in the fixed effect coefficients and confidence intervals between the two models, but the log likelihood was slightly higher for the model with random slopes, so this is the model we included in the paper. The random intercept fixed slope model is included in Additional file 1 (Table A2) for comparison. The random effects are reported with standard deviation instead of variance to make the interpretation more intuitive. The psychological distress variable was a little left and right skewed and a q-q plot indicated a decent fit for most of the range, but with some deviations for lower and higher values. Thus, we have added a sensitivity analysis with a mixed logistic regression model using a threshold for high psychological distress with mean item score of 1.85 in Additional file 1 (Table A3).

### Ethics

The Regional Ethical Committee for Medical Research in Western Norway approved the study (REK 2020/131560). Before responding to the email survey, all participants provided electronic informed consent; confidentiality and the right to withdraw from the study were guaranteed. The study adheres to the ethical criteria outlined in the Helsinki Declaration.

## Results

### Descriptive statistics

In the study population, 56% were female, 42% were under 50 years of age, 66% had a college- or university-level education, and 89% had a medium or high household income. Table 2 shows the distribution by age group.

**Table 2:** Baseline characteristics of the cohort

Age	18–29	30–39	40–49	50–59	60–69	70+
Male (%)	495 (34)	910 (39)	1176 (40)	1503 (42)	1625 (49)	1341 (53)
Educational level (%)						
Primary school	170 (12)	66 (3)	101 (4)	164 (5)	261 (8)	276 (11)
High school/trade school	456 (32)	364 (16)	541 (19)	1091 (31)	1047 (31)	802 (32)
College/University (short)	385 (27)	577 (25)	677 (23)	871 (24)	756 (23)	588 (23)
College/University (long)	408 (29)	1264 (56)	1575 (54)	1429 (40)	1225 (37)	858 (34)
Household income (%)						
Low	4218 (33)	223 (10)	256 (10)	242 (8)	166 (6)	241 (12)
Medium	470 (37)	1058 (48)	1417 (51)	1225 (37)	1022 (35)	1036 (50)
High	367 (30)	922 (42)	1103 (39)	1810 (55)	1682 (59)	770 (38)
High degree of psychological distress (%)	571 (40)	670 (29)	545 (18)	544 (15)	354 (11)	187 (7)
Physical activity (%)	1104 (76)	1823 (78)	2425 (82)	3103 (86)	2924 (88)	2191 (84)
Cultural practitioner (%)	343 (24)	484 (21)	634 (22)	1026 (29)	1119 (34)	864 (36)
Cultural audience (%)	445 (31)	855 (37)	993 (34)	999 (28)	978 (39)	964 (33)
Social networking (%)	429 (30)	543 (23)	860 (29)	1104 (31)	1059 (32)	1032 (31)
Volunteering and religious activities (%)	107 (7)	115 (5)	196 (7)	220 (6)	265 (8)	323 (13)

* *n* = 16,212. Primary school is ≤ 10 years of education, while college/university (short) is ≤ 3 years of higher education, and college/university (long) is > 3 years of higher education. Household income is based on the size of the family (first adult with weight 1, additional adults 0.70, and children 0.50). Norwegian krone (NOK) 250 K/year (Low), 250–500 K/year (Medium), and > 500 K/year (High); for Euros conversion use exchange rate on 21 November 2022. (10.4898). High degree of psychological distress = mean SCL-10 score ≥ 1.85. Cultural audience is defined as 1–3 times a month or more (before COVID-19), and the remaining activity dimensions are defined as once a week or more (before COVID-19). Information about recreational activities was collected at the second timepoint (t1), with retrospective questions about what the participants did before COVID-19.

### Activity dimensions and psychological distress

Table 3 shows the results from the linear mixed model testing the associations between the activity dimensions and psychological distress. In most groups, there were no substantial changes in psychological distress over time. At baseline, psychological distress was slightly lower among males than females (-0.037, CI -0.042; -0.032), and this gender difference persisted over time. When comparing age groups at baseline, psychological distress decreased with age, whereas for the 40–49 age group, there was a slight increase in psychological distress over time. We also find that the level of psychological distress at baseline decreased with increasing levels of education and income. For the higher income levels, we also observed a slight decrease in psychological distress over time.

In terms of the activity dimensions, people who had participated in physical activity weekly or more before COVID-19 had a lower level of psychological distress at baseline than those who had participated less frequently or not at all (-0.019, CI -0.026; -0.012), and this lower level of psychological distress did not significantly change over time. People who participated monthly or more as cultural audiences before COVID-19 had a slightly greater level of psychological distress at baseline (shortly after the COVID-19 restrictions were introduced) than those who participated less frequently or not at all in such activities before the pandemic (0.023, CI 0.017; 0.028). This initial higher level of psychological distress decreased over time (-0.004, CI -0.006; -0.001). For the other activity dimensions, there were no significant differences at baseline or over time.

The random effects show that there is a substantial variation between individual’s psychological distress at baseline, with individual intercepts varying 0.129 standard deviations (SD) from the mean. There is, however, little variation within the time trends of individuals (slope: SD 0.022). The covariance between individuals and time indicates that individuals with a higher level of psychological distress at baseline tended towards having a steeper slope over time.

**Table 3:** Degree of psychological distress and associations with participation in activity dimensions prior to COVID-19

Coefficients (95% confidence intervals)		
	Fixed effects	Time trend (per year)
Age		
18–29	0 (reference)	0 (reference)
30–39	-0.033 (-0.044; -0.022)	0.002 (-0.005; 0.008)
40–49	-0.083 (-0.093; -0.073)	0.008 (0.002; 0.013)
50–59	-0.098 (-0.108; -0.088)	-0.004 (-0.002; 0.010)
60–69	-0.122 (-0.133; -0.112)	-0.002 (-0.007; 0.004)
70+	-0.141 (-0.152; -0.130)	0.005 (-0.001; 0.011)
Sex		
Female	0 (reference)	0 (reference)
Male	-0.037 (-0.042; -0.032)	-0.002 (-0.005; 0.001)
Level of education		
Primary school	0 (reference)	0 (reference)
High school/trade school	-0.025 (-0.037; -0.014)	0.000 (-0.006; 0.007)
College/university (short)	-0.039 (-0.051; -0.027)	0.000 (-0.006; 0.007)
College/university (long)	-0.048 (-0.059; − 0.036)	0.000 (-0.007; 0.006)
Household income		
Low	0 (reference)	0 (reference)
Medium	-0.057 (-0.065; -0.048)	-0.005 (-0.010; 0.001)
High	-0.075 (-0.084; -0.066)	-0.008 (-0.012; -0.003)
Physical activity		
Less frequent	0 (reference)	0 (reference)
Weekly	-0.019 (-0.026; -0.012)	-0.001 (-0.004; 0.003)
Cultural practitioner		
Less frequent	0 (reference)	0 (reference)
Weekly	0.003 (-0.003; 0.009)	0.002 (-0.001; 0.005)
Cultural audience		
Less frequent	0 (reference)	0 (reference)
Monthly	0.023 (0.017; 0.028)	-0.004 (-0.006; -0.001)
Social networking		
Less frequent	0 (reference)	0 (reference)
Weekly	0.006 (-0.001; 0.012)	-0.001 (-0.005; 0.002)
Volunteering and religious activities		
Less frequent	0 (reference)	0 (reference)
Weekly	-0.008 (-0.018; 0.003)	0.003 (-0.002; 0.009)
Group	**Random effect**	**Standard deviation (95% confidence intervals)**
Individual	Intercept	0.129 (0.127; 0.131)
Time	Slope	0.022 (0.018; 0.027)
Time/individual	Covariance	0.125 (0.025; 0.222)
	Residual	0.085 (0.084; 0.087)

*Baseline constant of psychological distress: 0.365 (0.351; 0.380), time trend: 0.012 (0.004; 0.021). *n* = 14 333. Log likelihood = 22,125. Intraclass Correlation Coefficient (ICC) = 0.696 (0.685; 0.706). Linear mixed model presenting absolute coefficients with 0 indicating no difference/change, > 0 indicating a higher frequency of psychological distress within a group and < 0 indicating less psychological distress within a group. Psychological distress is a proportional scale from 0 to 1 where 0 is no psychological distress and 1 is the maximum level of psychological distress. As a sensitivity analysis, a mixed effects logistic regression model for high or low levels of psychological distress showed similar directions as the mixed linear model (Additional file 1, Table A3).

## Discussion

In this study, we evaluated the associations between participation in recreational activities before the pandemic and levels of psychological distress during the pandemic, measured at three time points, and adjusted for potential confounding factors such as sex, age, educational level, and household income. Our aim was to test whether the types of recreational activities people engaged in *before* the pandemic had any effect on levels of psychological distress *during* the pandemic. Using the pandemic as an “experiment” in this way could shed light on the importance of these activities to people’s mental health, as many of them were restricted during the pandemic, and/or whether engaging in these activities prior to a crisis could contribute to people’s mental resilience in any way.

Our findings provide valuable insights into the relationship between recreational activities and psychological distress during the COVID-19 pandemic. The results revealed a slightly higher level of psychological distress at baseline among people who frequently participated in cultural audience activities before COVID-19. Importantly, this was measured a few weeks into the lockdown period following the first pandemic measures toward COVID-19, which included the closing of cultural venues and cancellation or postponement of all cultural events. Thus, the elevation in psychological distress for this group could be linked to the immediate impact of these lockdown measures. Furthermore, time trends indicated that those who participated as cultural audience monthly or more before the pandemic seemed to reduce their levels of psychological distress over time, more so than those who participated less frequently or not at all. These results suggest the importance of cultural activities for people’s mental health, revealing how psychological distress increases when activities are restricted and decreases again when they are reintroduced. This finding could also suggest that these types of activities have a protective effect on people’s mental health over time, echoing findings in other studies where cultural audience participation is positively associated with life satisfaction, as well as a reduction in mental distress, anxiety and depression [9, 12]. However, we cannot exclude the possibility of a third factor causing this pattern. Many phenomena could fluctuate, and when measuring these at a time when they are more pronounced or extreme, as in our case with higher levels of psychological distress, the fluctuation itself will contribute to a higher likelihood of measuring less pronounced or extreme measures at a later time point (regression to the mean).

Those who participated regularly in physical activities before the pandemic had lower levels of psychological distress at baseline. There was no significant change in psychological distress over time for this group, which might indicate that these types of activities were not particularly affected by pandemic restrictions and/or that physical activities might have helped maintain lower distress levels, functioning as a protective mechanism over time [8]. This further cements the link between physical activities and mental health observed in other studies, both in [31, 32] and outside of the pandemic context [33, 34].

Interestingly, we do not find any clear associations between the other dimensions of recreational activities (cultural practitioner, social networking, and volunteering and religious activities) and levels of psychological distress at baseline or over time. This contrasts with findings from other studies, such as the Norwegian HUNT Study, which revealed a significant association between positive mental health outcomes and participation in both receptive (audience) and creative (practical) cultural participation [12]. In the social networking dimension, we have grouped organized activities you would do together with people who are not necessarily your closest friends or relatives to assess whether this social aspect has any association with psychological distress. Surprisingly, we found no such associations in our analysis. This might indicate that these types of activities were easier to perform or find substitutes for during the pandemic than for that of cultural audience activities. Cultural audience activities were more directly impacted by the closing of institutions, even though some institutions did broadcast events online.

Linking the term collective effervescence to our study provides an interesting way to look at our findings around cultural audience participation. Cultural activities, such as attending a concert, theatre performance, museum, or cinema, evoke a sense of collective effervescence, as they bring audiences together in communal experiences of creative expression. In these moments, the boundary between individuals and the collective blurs, as people connect through the shared appreciation of culture and art, functioning as vehicles to reinforce social cohesion [35], which might not be as easily attained through attending these events online. Moreover, engaging in activities such as art, music, or sports helps individuals build social ties, which are essential for emotional support and a sense of belonging, aspects that are also inherent in collective coping [17]. During a crisis such as the COVID-19 pandemic, access to these types of activities was restricted over periods of time, and this seemingly had a significant impact on levels of psychological distress.

Our study has both strengths and limitations. A major strength of our study is that it includes a large sample of individuals (16,212 at baseline). These were followed up at three time points over a two-year period, from the initial phase of the pandemic in Norway until after the population was fully vaccinated and all pandemic restrictions were ended. As such, this is one of few longitudinal studies looking at recreational activities and mental health outcomes in the context of the pandemic.

There was, however, a high number of lost-to-follow-up during the two-year period with approximately half of the initial population followed up at the last time point. This introduces potential selection bias, as those with higher psychological distress might have been lost to a higher degree than those with less psychological distress. There could also be a non-response bias that participants with higher levels of psychological distress to lower degree has responded to any of the time points. Additionally, it is possible that those who engaged in regular physical or cultural activities pre-pandemic generally had healthier lifestyles, which could independently contribute to lower psychological distress. Selection bias might also result from certain recreational activities (e.g., cultural audience participation) being more accessible to individuals with higher socioeconomic status. Moreover, since the survey was conducted online, individuals with lower digital literacy or immigrants with limited proficiency in the survey language (Norwegian) may be underrepresented. Another potential limitation is that questions about recreational activities engaged in before the pandemic were asked retrospectively at the second time point (January 2021), which might have resulted in under- or overreporting of activities. Lastly, the linear mixed model regression was not optimal in terms of model fit, as the distribution of psychological distress was both left and right skewed. To remedy this, we added a dichotomized model using a mixed effects logistic regression model as a sensitivity analysis (see Table A3 in Additional file 1). This showed comparable directions in the coefficients as the continuous model, thus confirming our original findings.

## Conclusions

Overall, the study highlights the importance of not only physical activities but also participation in cultural audience activities, such as concerts, cinema, theatre and going to the library, in preventing psychological distress. This finding reinforces the link between recreational activities and mental health, suggesting that engaging in these activities can contribute to mental resilience during crises.

## Electronic supplementary material

Below is the link to the electronic supplementary material.

Supplementary Material 1

## Data Availability

The data presented in this study are available on request from the corresponding author.

## Abbreviations

COVID-19: Coronavirus disease 2019
SCL-10: Hopkins Symptoms Checklist 10
HUNT-study: The Trøndelag Health Study
