# Supplementary material for: Recreational activities and psychological distress during the COVID-19 crisis: a cohort study from Norway
Source: BMC Public Health. 2025 May 31;25:2020. doi: 10.1186/s12889-025-22942-7 (PMC12125845; doi:10.1186/s12889-025-22942-7)
Supplement: Supplementary file 1 — Supplementary Material 1 [file 12889_2025_22942_MOESM1_ESM.pdf]

Additional file 1

To research article: Recreational activities and psychological distress during the COVID-19 crisis: A cohort study from Norway

Table A1: Description of survey items used in this study in full.

TIME-POINT	QUESTIONS AND ANSWER ALTERNATIVES					
T1	**How often did you do the following activities in your spare time before the COVID-19 pandemic?**					
		Did not do this before COVID-19	Less than once a month	1-3 times a month	Every week	Daily
	Organized sports	x	x	x	x	x
	Individually organized sports/exercise	x	x	x	x	x
	Going for a walk	x	x	x	x	x
	Organized song, music, theater, or dance	x	x	x	x	x
	Other organized cultural activities	x	x	x	x	x
	Hobby activity/arts and crafts done alone or with friends	x	x	x	x	x
	Going to a museum or an arts exhibition	x	x	x	x	x
	Going to a concert or theater performance	x	x	x	x	x
	Going to the cinema	x	x	x	x	x
	Going to the library	x	x	x	x	x
	Participate in volunteer work in health, social work or the like	x	x	x	x	x
	Participate in activities in a religious community, including religious meetings	x	x	x	x	x
T0/T1/T2	**Think back on the last 7 days and chose the alternative that reflects your experience. Answer as honest as possible.**					
		Not bothered	Slightly bothered	Quite bothered	Very bothered	
	Sudden fear without specific cause	x	x	x	x	
	Feeling scared or anxious	x	x	x	x	
	Fatigue or dizziness	x	x	x	x	
	Feeling tense or agitated	x	x	x	x	
	Easily blame yourself	x	x	x	x	
	Problems sleeping	x	x	x	x	
	Feeling useless, worthless	x	x	x	x	
	Depressed, melancholic (sad)	x	x	x	x	

	Feeling that everything is a struggle	x	x	x	x	
	Feeling hopeless about the future	x	x	x	x	
**T0**	**What is your highest completed level of education?**					
	Primary education up to 10 years	x				
	High school/trade school	x				
	Higher education, 3 years or less	x				
	Higher education, 4 years or more	x				
**T0**	**Approximately what was your household's total gross income last year? Include all income from work and benefits.**					
	Under 100 000 NOK	x				
	100 000 NOK	x				
	200 000 NOK	x				
	…	x				
	1 900 000 NOK	x				
	2 000 000 NOK or more	x				
**T0**	**Age category**					
	18-29	x				
	30-39	x				
	40-49	x				
	50-59	x				
	60-69	x				
	70+	x				
**T0**	**Sex**					
	Male	x				
	Female	x				

* The survey also includes other questions included in other studies, but these will not be described here.

Table A2: Alternative mixed effects model with fixed slopes, random intercept (group: individual). Degree of psychological distress and associations with participation in activity dimensions prior to COVID-19

Coefficients (95% confidence intervals)		
	Fixed effects	Time trend (per year)
**Age**		
18-29	0 (reference)	0 (reference)
30-39	-0.033 (-0.044; -0.022)	0.002 (-0.004; 0.008)
40-49	-0.083 (-0.094; -0.072)	0.008 (0.002; 0.014)
50-59	-0.098 (-0.108; -0.088)	-0.004 (-0.001; 0.010)
60-69	-0.122 (-0.133; -0.112)	-0.001 (-0.007; 0.004)
70+	-0.141 (-0.153; -0.130)	0.005 (-0.001; 0.011)
**Sex**		
Female	0 (reference)	0 (reference)
Male	-0.037 (-0.042; -0.032)	-0.002 (-0.005; 0.001)
**Level of education**		
Primary school	0 (reference)	0 (reference)
High school/trade school	-0.026 (-0.038; -0.014)	0.001 (-0.006; 0.007)
College/university (short)	-0.039 (-0.051; -0.027)	0.000 (-0.006; 0.007)
College/university (long)	-0.048 (-0.060; - 0.036)	0.000 (-0.006; 0.006)
**Household income**		
Low	0 (reference)	0 (reference)
Medium	-0.057 (-0.066; -0.048)	-0.005 (-0.010; 0.000)
High	-0.075 (-0.084; -0.066)	-0.007 (-0.012; -0.002)
**Physical activity**		
Less frequent	0 (reference)	0 (reference)
Weekly	-0.019 (-0.026; -0.012)	-0.001 (-0.004; 0.003)
**Cultural practitioner**		
Less frequent	0 (reference)	0 (reference)
Weekly	0.003 (-0.003; 0.009)	0.002 (-0.001;0.006)
**Cultural audience**		
Less frequent	0 (reference)	0 (reference)
Monthly	0.023 (0.017; 0.028)	-0.004 (-0.006; -0.001)
**Social networking**		
Less frequent	0 (reference)	0 (reference)
Weekly	0.006 (-0.001; 0.012)	-0.001 (-0.005; 0.002)
**Volunteering and religious activities**		
Less frequent	0 (reference)	0 (reference)
Weekly	-0.008 (-0.019; 0.003)	0.003 (-0.002; 0.009)
**Group**	**Random effect**	**Standard deviation (95 % confidence intervals)**
Individual	Intercept	0.132 (0.130; 0.133)
	Residual	0.088 (0.087; 0.089)

*Baseline constant of psychological distress: 0.366 (0.351; 0.381), time trend: 0.011 (0.003; 0.019). n = 14 333. Log likelihood = 22079. Intraclass Correlation Coefficient (ICC) = 0.692 (0.684; 0.699). Linear mixed model presenting absolute coefficients with 0 indicating no difference/change, >0 indicating a higher frequency of psychological distress within a group and <0 indicating less psychological distress within a group.

Klasse: Åpen

Table A3: Sensitivity analysis: Mixed effects logistic regression model for degree of psychological distress (high/low) and associations with participation in activity dimensions prior to COVID-19

Coefficients (95% confidence intervals)		
	Fixed effects	Time trend (per year)
**Age**		
18-29	0 (reference)	0 (reference)
30-39	-0.582 (-0.897; -0,268)	0.050 (-0.161; 0,261)
40-49	-1.900 (-2.218; -1.581)	0.297 (0.088; 0,506)
50-59	-2.275 (-2.592; -1.958)	0.157 (-0.049; 0.363)
60-69	-2.994 (-3.340; -2.648)	-0.019 (-0.242; 0.204)
70+	-3.877 (-4.285; -3,469)	0.222 (-0034; 0.477)
**Sex**		
Female	0 (reference)	0 (reference)
Male	-0.924 (-1.105; -0.743)	-0.052 (-0.173; 0.068)
**Level of education**		
Primary school	0 (reference)	0 (reference)
High school/trade school	-0.507 (-0.885; -0.130)	-0.040 (-0.299; 0.218)
College/university (short)	-0.922 (-1.307; -0.538)	-0.019 (-0.280; 0.241)
College/university (long)	-1.331 (-1.709; -0.953)	-0.003 (-0.258; 0.252)
**Household income**		
Low	0 (reference)	0 (reference)
Medium	-1.176 (-1.447; -0.905)	-0.158 (-0.336; 0.019)
High	-1.671 (-1.954; -1.388)	-0.214 (-0.398; -0.030)
**Physical activity**		
Less frequent	0 (reference)	0 (reference)
Weekly	-0.424 (-0.650; -0.198)	-0.023 (-0.175; 0.129)
**Cultural practitioner**		
Less frequent	0 (reference)	0 (reference)
Weekly	0.186 (-0.015; 0.387)	-0.031 (-0.163; 0.101)
**Cultural audience**		
Less frequent	0 (reference)	0 (reference)
Monthly	0.571 (0.388; 0.753)	-0.142 (-0.262; -0.022)
**Social networking**		
Less frequent	0 (reference)	0 (reference)
Weekly	0.090 (-0.126; 0.305)	0.069 (-0.074; 0.212)
**Volunteering and religious activities**		
Less frequent	0 (reference)	0 (reference)
Weekly	-0.201 (-0.573; 0.171)	0.080 (-0.162; 0.322)
**Group**	**Random effect**	**Variance (95 % confidence intervals)**
Individual	Intercept	12.142 (10.847; 13.591)

*Baseline constant of psychological distress: 1.174 (0.714; 1.635), time trend: 0.339 (0.025; 0,654). n = 14 333. Log likelihood = -13415. Intraclass Correlation Coefficient (ICC) = 0.787 (0.767; 0.805). Psychological distress is a dichotomous variable (low = 0 / high = 1) made from an index variable (SCL10) coded 1-4. Threshold for low psychological distress was set to <1.85, high was set to >=1.85 (see methods section for more on threshold).
